# Identification of Melioidosis Outbreak by Multilocus Variable Number Tandem Repeat Analysis 

**DOI:** 10.3201/eid1502.081036

**Published:** 2009-02

**Authors:** Bart J. Currie, Asha Haslem, Talima Pearson, Heidie Hornstra, Benjamin Leadem, Mark Mayo, Daniel Gal, Linda Ward, Daniel Godoy, Brian G. Spratt, Paul Keim

**Affiliations:** Menzies School of Health Research, Darwin, Northern Territory, Australia (B.J. Currie, A. Haslem, M. Mayo, D. Gal, L. Ward); Northern Territory Clinical School, Darwin (B.J. Currie, L. Ward); Northern Arizona University, Flagstaff, Arizona, USA (T. Pearson, H. Hornstra, B. Leadem, P. Keim); Imperial College, London, UK (D. Godoy, B.G. Spratt)

**Keywords:** Burkholderia pseudomallei, melioidosis, outbreak, VNTR, MLVA, MLST, phylogenetic analysis, research

## Abstract

Endemic melioidosis is caused by genetically diverse *Burkholderia pseudomallei* strains. However, clonal outbreaks (multiple cases caused by 1 strain) have occurred, such as from contaminated potable water. *B. pseudomallei* is designated a group B bioterrorism agent, which necessitates rapidly recognizing point-source outbreaks. Pulsed-field gel electrophoresis (PFGE) and multilocus sequence typing (MLST) can identify genetically related isolates, but results take several days to obtain. We developed a simplified 4-locus multilocus variable number tandem repeat analysis (MLVA-4) for rapid typing and compared results with PFGE and MLST for a large number of well-characterized *B. pseudomallei* isolates. MLVA-4 compared favorably with MLST and PFGE for the same isolates; it discriminated between 65 multilocus sequence types and showed relatedness between epidemiologically linked isolates from outbreak clusters and between isolates from individual patients. MLVA-4 can establish or refute that a clonal outbreak of melioidosis has occurred within 8 hours of receipt of bacterial strains.

## Abstract 2

One-sentence summary for table of contents: This analysis can identify a clonal outbreak of this disease within 8 hours of receipt of bacterial isolates.

Melioidosis is endemic in Southeast Asia and northern Australia (*1**,**2*). The reported incidence of melioidosis has been increasing within this region, and new foci and outbreaks of melioidosis are being described within this region and in distant locations such as Brazil (*3*) and New Caledonia (*4*). It remains unclear how much of this expansion of the global distribution boundaries is from recent spread of the causative bacterium, *Burkholderia pseudomallei*, and how much is from unmasking of disease after events such as the 2004 Asian tsunami (*5**,**6*). Molecular studies have shown considerable genetic diversity within *B*. *pseudomallei* (*1**,**2**,**7*). For instance, in northern Australia, isolates from patients are generally distinct from each other (*8*) unless there is a point-source outbreak, such as occurred in 2 episodes after *B*. *pseudomallei* contamination of community water supplies (*9**,**10*).

In disease-endemic regions, melioidosis case numbers surge in the monsoonal wet season, and individual cases are typically caused by different *B*. *pseudomallei* strains. However, under some circumstances, a series of cases can be caused by 1 strain, indicating that a clonal or point-source outbreak has possibly occurred and an urgent public health response may be required. Because several days are required to perform the currently available molecular typing methods of ribotyping, multilocus sequence typing (MLST) and pulsed-field gel electrophoresis (PFGE), the ability to rapidly distinguish endemic infection from a clonal outbreak has not been possible. Furthermore, *B*. *pseudomallei* is classified as a group B bioterrorism agent by the US Centers for Disease Control and Prevention, and the implications of a possible deliberate release warrant the availability of a robust method to quickly ascertain if concomitant cases of melioidosis are caused by 1 bacterial strain.

We recently described using a BOX-PCR for rapid typing of *B*. *pseudomallei* (*11*). We have now adapted and simplified multilocus variable number tandem repeat (VNTR) analysis (MLVA) for rapid typing because this analysis potentially enables precise international strain comparisons. We have compared MLVA results on a wide range of well-characterized *B. pseudomallei* isolates with those for MLST and PFGE.

## Methods

### MLVA, PFGE, and MLST

U’Ren et al. initially described 32 VNTR loci for *B*. *pseudomallei* that had 7–28 alleles (*12*). Thirty of these VNTR markers were subsequently used for fine-scale analysis of 121 isolates of *B*. *pseudomallei* (*13*). Various combinations of markers were tested by MLVA; we chose 4 markers that were highly discriminatory, enabling single-run, 4-color analysis in a DNA sequencer. The 4 VNTR loci chosen were 2341, 389, 1788, and 933 (*12*). Table 1 shows the PCR primers used and the repeat region amplified for each locus. VNTR loci 2341 and 933 are from *B*. *pseudomallei* chromosome 1, and loci 389 and 1788 are from chromosome 2.

**Table 1:** Characteristics of 4 VNTR loci used for identification of Burkholderia pseudomallei*

Characteristic	VNTR loci			
	2341	1788	933	389
Color-labeled forward primer sequence (5′ → 3′)	FAMGGCTTCGCACCCGCCCCATTTCAGC	PETGCGCGGCGAGAACGGCAAGAACGAA	NEDATGGTGGCGGCCGTCGGCGAAAACC	VICGTTACAAGCGCGGGTCGGCAAGAGGCTGAAA
Reverse primer sequence (5′ → 3′)	GCACCGGGCGCGGCGCACTCG	GAGCATCGGGTGGGCGGCGCGTATTGAT	GCTCGAATGGGTGTACGAAGGGCCACGCTGATTC	GCCGGTGTTGAACGAGTGGGTGGCGTAAGC
Repeat sequence (5′ → 3′)	TTCGTGCGC	GTCGTGCGATCCTGCT	CGGCGAGGGAAA	GACGAACC
Minimum size, bp† (no. repeats)	111 (2)	235 (4)	171 (3)	221 (1)
Maximum size, bp (no. repeats)	243 (17)	382 (13)	337 (17)	292 (10)
No. alleles	16	10	13	9
No. null alleles	–	–	2/65 STs	–

*VNTR, variable number tandem repeat; STs, sequence types. †Error range in fragment sizing is ± 3 bp.

PCRs contained 0.88 U HotStarTaq DNA Polymerase (QIAGEN, Hilden, Germany) per reaction, 1× PCR buffer, 1.2 M Betaine, 3 mmol/L MgCl_2_, 0.2 mmol/L deoxynucleoside triphosphates, 0.2 μM fluorescently labeled forward primer, 0.2 μM reverse primer, 1 μL template DNA (0.5 ng/μL), and double-distilled water to give a volume of 11 μL per reaction. Amplifications were conducted in Palm Cyclers (Corbett Research, Sydney, New South Wales, Australia). All PCRs underwent initial denaturation at 95°C for 5 min, then 34 cycles of 94°C for 30 s, 68°C for 30 s, and 72°C for 30 s, followed by a final extension step of 72°C for 5 min and 15°C for 3 min.

PCR products of each colored primer (FAM, NED, PET, and VIC; Table 1) were then pooled. Pooled PCR products were diluted with 200 μL of double-distilled water, and 1.2 μL of PCR product was added to a mixture of a 1:6 ratio of Hi-Di formamide (Applied Biosystems, Foster City, CA, USA) and GeneScan 1200 LIZ (Applied Biosystems) fluorescently labeled size standard. PCR products were then electrophoretically separated by using a 3100xl DNA Sequencer (Applied Biosystems) and analyzed by using the ABI software program GeneMapper version 3.5 (Applied Biosystems). PFGE with *Spe*I and MLST were performed as described (*7**,**14*).

### Data Analysis

For 4-locus multilocus VNTR analysis (MLVA-4), GeneMapper peak files were imported into BioNumerics version 4.61 (Applied Maths BVBA, Sint-Martens-Latem, Belgium). Relatedness of isolates was assessed by using a matrix of the pairwise differences of the 4 VNTR loci, with a dendrogram produced by using the unweighted pair group method with arithmetic averages (UPGMA).

For PFGE, gel images were analyzed with BioNumerics version 4.61. BioNumerics application modules used were Fingerprint Types and Comparison and Cluster Analysis modules. PFGE bands (150–700 kbp) were manually assigned on visual inspection. PFGE dendrograms were produced with Dice UPGMA with position tolerance settings of 0.5% optimization and 1.0% band position tolerance.

For MLST, alleles at each of the 7 previously described loci (*7*) were assigned for each isolate by comparing sequences to those at the *B*. *pseudomallei* MLST website (*15*). Following the standard MLST protocol, each allele was assigned a different allele number, and the allelic profile (string of 7 integers) was used to define the sequence type (ST) for that isolate. Allelic profiles of isolates were imported into BioNumerics version 4.61, and relatedness of isolates was displayed as a dendrogram by using the matrix of pairwise differences in the allelic profiles and UPGMA clustering.

### *B. pseudomallei* Isolates

To assess the discriminatory power of MLVA-4, direct comparisons were made between the MLST dendrogram for 65 *B*. *pseudomallei* isolates, each representing a distinct ST, and the MLVA-4 dendrogram for these isolates. The 65 isolates were all from Australia and included human, animal, and environmental sources. There were 16 pairs of single-locus variants (SLVs; isolates sharing identical alleles at 6/7 loci by MLST).

To assess the ability of MLVA-4 to identify clonal clusters, direct comparisons were made between the PFGE dendrogram for 4 defined clonal groups and the MLVA-4 dendrogram for these isolates. Clonal cluster I and clonal cluster II consist of 8 and 7 isolates, respectively, from the tropical Northern Territory of Australia and were previously identified as clustering by PFGE (*16*). These 2 clonal clusters represent geographically linked but epidemiologically unrelated isolates from our prospective melioidosis studies in northern Australia. Clonal cluster III consists of 3 isolates of identical ST cultured from a detergent container implicated in an outbreak of melioidosis in Northern Territory involving 2 garage mechanics (*14*). Clonal cluster IV contains 6 isolates (5 from humans, 1 from water) from an outbreak of melioidosis in a remote indigenous community in Northern Territory. The outbreak was linked to contamination of the unchlorinated community water supply, with several deaths reported (*10*).

**Figure 1:**
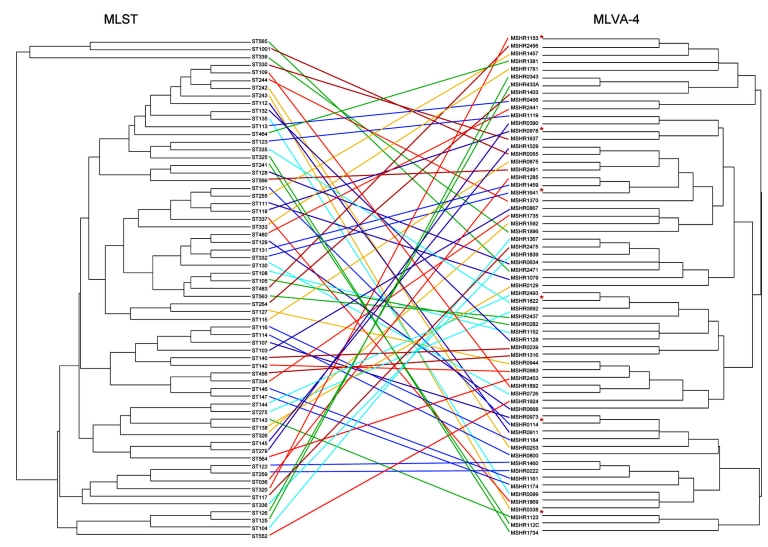
Comparison of multilocus sequence typing (MLST) and 4-locus multilocus variable number tandem repeat analysis (MLVA-4) dendrograms for 65 *Burkholderia pseudomallei* isolates. MLST sequence type (ST) is shown for each isolate, with the corresponding isolate number listed for the MLVA-4 profile and shown by the colored lines. The red asterisks indicate 6 isolates that represent diversity of MLVA-4; these isolates were used to calibrate the dendrogram in Figure 3.

Finally, to assess the ability of MLVA-4 to link isolates from patients, we analyzed multiple isolates from 3 patients. Patient A had chronic pulmonary melioidosis, and 5 *B*. *pseudomallei* isolates were recovered over 22 months. Patient B had chronic pulmonary melioidosis, and 7 isolates were recovered over 6 years, including 2 isolates from this patient’s water supply. Patient C died of melioidosis septicemia; 6 isolates were recovered over 14 days. To construct the dendrogram for these clinical isolates, we chose 6 unrelated isolates representing the diversity of Australian isolates seen with MLVA-4 (Table 2). These 6 isolates are indicated in Figure 1. This study was reviewed and approved by the Human Research Ethics Committee of the Northern Territory Department of Health and Community Services and the Menzies School of Health Research, Darwin, Northern Territory, Australia (approval 02/38).

**Table 2:** Fragment size and repeat copy number (MLVA-4 code) for 6 *Burkholderia pseudomallei* strains used as MLVA-4 standards*

Strain	VNTR loci										
	2341			389			1788			933	
	Size, bp†	Repeat copy no.		Size, bp†	Repeat copy no.		Size, bp†	Repeat copy no.		Size, bp†	Repeat copy no.
MSHR978	189.85	11		236.19	8		265.63	6		254.8	10
MSHR1822	190.25	11		245.02	9		282.56	7		290.55	13
MSHR114	145.49	6		252.32	10		298.4	8		242.57	9
MSHR1641	154.5	7		236.22	8		315.39	9		230.81	8
MSHR1153	127.93	4		236.24	8		298.51	8		194.96	5
MSHR1123	172.34	9		260.28	11		331.48	10		218.91	7

*MLVA-4, 4-locus multilocus variable number tandem repeat analysis. †Error range in fragment sizing is ± 3 bp.

## Results

Table 1 shows size variation with calculated number of repeats and number of alleles for each of the 4 VNTR loci. Locus 933 showed null alleles for 2 of the 65 MLST STs.

Figure 1 shows the relationship between the 65 discrete MLST STs and the MLVA-4 for these isolates. MLVA-4 was able to discriminate between each ST. Relationships between STs seen on the MLST dendrogram were generally not preserved with MLVA-4. This is expected because VNTRs change too rapidly and too few loci were used to compensate for homoplasy at individual loci and to provide phylogenetic content to the assay. However, strains that were closely related by MLST (SLVs) could in some cases be seen to be related by using MVLA-4 (Figure 1).

**Figure 2:**
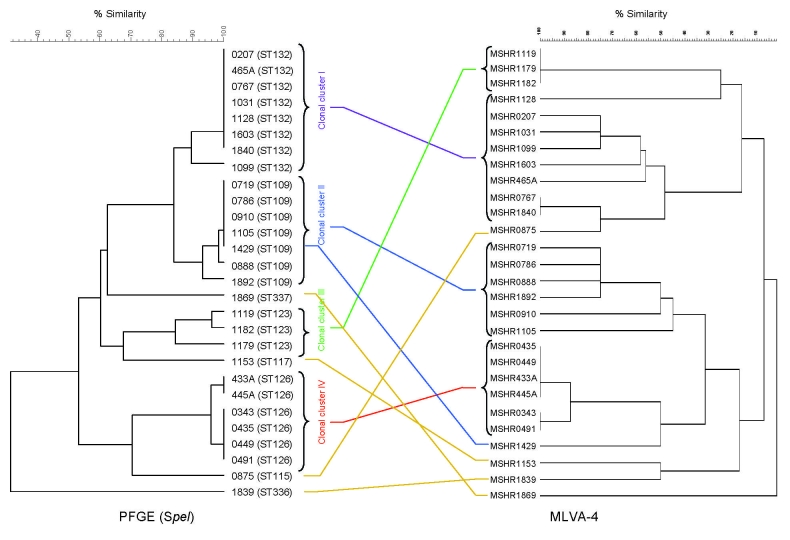
Comparison of pulsed-field gel electrophoresis (PFGE) and 4-locus multilocus variable number tandem repeat analysis (MLVA-4) profiles for isolates in 4 clonal groups (see text for details). Isolate number with its MLST sequence type (ST) is listed for each isolate on the PFGE profile, with the corresponding isolate number listed for the MLVA-4 profile. Four unrelated isolates are included for comparison: 0875 (ST115), 1869 (ST337), 1839 (ST336), and 1153 (ST117).

Figure 2 shows results for the 24 isolates in the cluster study, with 4 additional unlinked isolates, each from a different ST included for comparison. There was generally excellent agreement between PFGE and MLVA-4 for each of the 4 clonal clusters. PFGE clonal clusters I (MLST ST 132) and II (ST 109), each containing epidemiologically unrelated strains, also clustered on MLVA-4, with the exception of isolate MSHR1429, which by MLVA-4 was located outside its cluster group. The detergent cluster III (ST 123) was indistinguishable by MLVA-4, and the community outbreak strains in cluster IV (ST 125, ST 126) separated into 2 closely linked MLVA-4 patterns, 1 of which included the isolate from the community water supply (MSHR491, ST 126).

**Figure 3:**
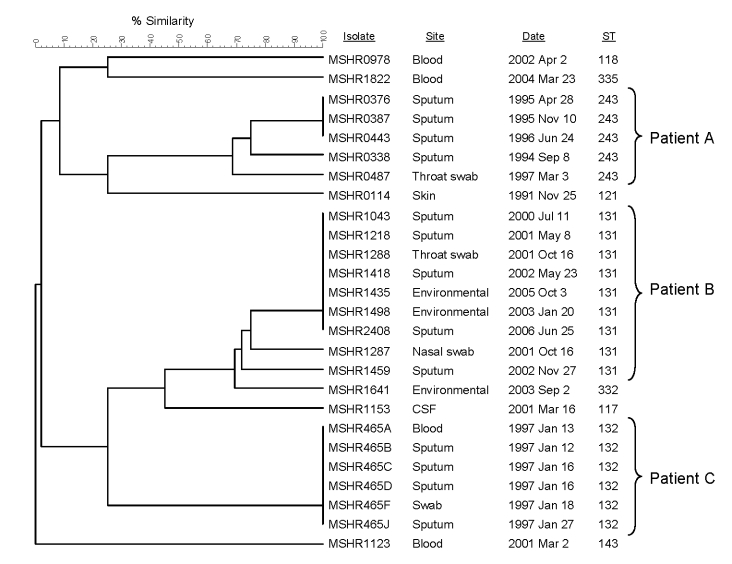
Dendrogram showing 4-locus multilocus variable number tandem repeat analysis profiles for isolates from 3 patients with melioidosis, with isolate number and multilocus sequence typing sequence type (ST) listed (see text for details). Six isolates used to calibrate the dendrogram are indicated by asterisks in Figure 1 and listed in Table 2. CSF, cerebrospinal fluid.

Figure 3 shows MLVA-4 results for the 3 patients. Isolates from patient A (ST 243) and B (ST 131) with chronic pulmonary melioidosis each had closely linked MLVA-4 results with a suggestion of fine-scale differentiation over the years of infection. The 2 water supply isolates from patient B were identical to 5 of her clinical isolates. The 6 clinical isolates from patient C, who had fatal melioidosis, were identical by MLVA-4, including isolates from blood and sputum.

## Discussion

Ribotyping was the first method widely used for typing *B*. *pseudomallei* (*17*), followed by PFGE. To date, PFGE has been considered the standard method for investigating potential point-source outbreaks of bacterial infections. We have previously used PFGE to link case clusters of melioidosis to water supply contamination (*10*) and to contamination of a container of detergent (*14*). However, such outbreaks are rare, and we have shown that, in the melioidosis-endemic region of northern Australia, case clusters during extreme weather events are usually not genetically linked by PFGE (*8*). These clusters simply reflect the close association between rainfall and infection from the diverse range of *B*. *pseudomallei* strains in soil and surface water.

Recently, MLST has enabled new insights into regional and global epidemiology of melioidosis (*7**,**16**,**18**–**20*). Although there is excellent congruence between PFGE and MLST, with PFGE and MLST providing similar results for local epidemiologic investigations (*16*), MLST has the major advantage of absolute comparative ability across laboratories through the MLST website and unambiguous sequence type characterization.

Ribotyping and PFGE take several days to generate results, and MLST is expensive and requires sequencing and analysis capability. PCR-based typing methods have enabled more rapid availability of results. Randomly amplified polymorphic DNA (RAPD) analysis has been used to analyze relationships between clinical and environmental *B*. *pseudomallei* (*21**,**22*). However, it is not possible to make valid comparisons of RAPD results between laboratories and sometimes even between runs in the same laboratory. Thus, despite the speed of RAPD, we no longer use it.

Analyzing bacterial genomes for VNTRs has enabled MLVA assays to be developed to differentiate among methicillin-resistant *Staphylococcus aureus* strains that are indistinguishable by PFGE (*23*) and to differentiate *Neisseria meningitidis* strains with identical MLST STs (*24*). Liu et al. developed the first MLVA system for *B*. *pseudomallei* (*25*). They selected 5 VNTR loci from the *B*. *pseudomallei* genome to include in a multiplex PCR–based MLVA that enabled them to demonstrate extensive diversity among 32 *B*. *pseudomallei* strains obtained during an unprecedented 4-month increase in melioidosis cases in Singapore in early 2004. Their results clearly excluded a point-source outbreak and suggested that the case cluster was related to the particularly high rainfall that occurred that year.

*B*. *pseudomallei* contains numerous VNTRs. Using a 32 VNTR system, U’Ren et al. showed extensive diversity within a global *B*. *pseudomallei* isolate set (*26*). When 30 of these VNTR loci were used to analyze 9 epidemiologically related *B*. *pseudomallei* isolate sets, fine-scale diversity was found even among closely related strains, including sequential isolates from persons (*13*). We sought to develop a rapid and robust minimum loci *B*. *pseudomallei* MLVA that differentiated unrelated strains and maintained the ability to link isolates from a point-source outbreak. Our approach was similar to that developed for MLVA of *N*. *meningitides*, in which an 8-locus system was used to look at the global epidemiology, with clustering similar to that obtained with MLST. In this system, 4 highly variable VNTR loci were then chosen to analyze *N*. *meningitidis* serogroup C strains collected during a meningococcal outbreak in the Netherlands (*24*).

Our 4-locus MLVA for *B*. *pseudomallei* separated all 65 MLST STs analyzed. In addition to being highly discriminatory, the MLVA-4 had good specificity in clustering genetically linked *B*. *pseudomallei* strains and performed as well as PFGE in identifying clonal clusters. In particular, MVLA-4 could distinguish between epidemiologically unlinked strains that were identical by MLST and PFGE (groups I and II; Figure 2), while isolates from confirmed point-source outbreaks (groups III and IV; Figure 2) were either identical or closely clustered. Similarly, multiple isolates from a patient with acute disease obtained over 2 weeks were all identical (patient C; Figure 3), and those recovered over a much longer period from patients with chronic disease were closely clustered but showed some diversification (patients A and B, chronic disease over years; Figure 3).

Because PFGE takes >5 days to obtain results, alternative typing methods are required to rapidly determine whether a cluster of melioidosis cases is genetically linked and therefore potentially an outbreak that requires an urgent public health response. We recently demonstrated that BOX-PCR can perform similarly to PFGE and MLST in typing *B*. *pseudomallei*, with the ability to usually discriminate between unrelated isolates, while also showing relatedness of epidemiologically linked isolates (*11*). However, although BOX-PCR can provide results within 10 hours of a laboratory receiving the bacterial strains, it is less reproducible than PFGE, and a reliable comparison of BOX-PCR results between laboratories is not possible (*27*). We found variation in BOX-PCR results when we compared results from different PCR machines in our own laboratory and band-density differentials dependent on DNA template concentration (*11*).

MLVA-4 results are generally reproducible and can be obtained quickly (*24*). In the initial *B*. *pseudomallei* MLVA used to investigate the Singapore cluster, agarose gel electrophoresis was used to size multiplexed PCR products and enabled analysis on the basis of the VNTR banding profile (*25*). However, use of a DNA sequencer for simultaneous sizing of the 4 fluorescently labeled PCR products enables >16 isolates to be analyzed in 1 run with our MLVA-4, and results are potentially available 8 hours after receipt of bacterial strains. For related but not identical MLVA-4 patterns, we assessed the specificity of strain clustering by generating dendrograms that compared strains in question with 6 reference strains that represented the considerable diversity seen on MLVA-4 (Figures 1, 3). Table 2 provides fragment size and repeat copy number (MLVA-4 code) data on these 6 strains for use as standards by other laboratories in generating their own MLVA-4 results for their own *B*. *pseudomallei* strains, with potential for direct comparison of MLVA-4 results between different laboratories. Subsequently, MLST can be used to verify relatedness of strains through the MLST database.

In summary, we have developed a simplified 4-locus MLVA that compares favorably with PFGE and MLST. This analysis can be used to recognize or exclude a point-source outbreak of melioidosis within 8 hours of receipt of *B*. *pseudomallei* strains.
